# REMOTE TELEASSESSMENT AND TELEREHABILITATION OF A COMPREHENSIVE EXERCISE TRAINING PROTOCOL FOR OLDER ADULTS

**DOI:** 10.1093/geroni/igac059.2236

**Published:** 2022-12-20

**Authors:** Savitha Subramaniam, Edwina Wilson, Tanvi Bhatt

**Affiliations:** University of Illinois at Chicago, Chicago,, Illinois, United States; University of Illinois at Chicago, Chicago, Illinois, United States; University of Illinois at Chicago, Chicago,, Illinois, United States

## Abstract

**Background:**

With the increased implementation of interactive technologies for assessment and rehabilitation, it would be optimal to exhibit the reliability of physical assessment measures via tele-assessment. Aim: To determine the test-retest and intra-rater reliability of physical function outcome measures routinely used in the balance and gait rehabilitation using real-time online tele-assessment.

**Methods:**

Community-dwelling healthy older adults (N=30) participated in three experimental tele-assessment sessions. During each session, a real-time online tele-assessment was performed on five major domains that evaluate balance and gait function: lower limb strength and endurance (30-second chair stand test), aerobic endurance (2-minute step test), static balance (One-legged stand test), dynamic balance (4-step square test), and gait (Tinetti).

**Results:**

Coefficient of determination (R2) was used to determine the test-retest (TRT) and intra-rater (IR) reliability for all outcome variables. Excellent reliability was shown by Tinetti (TRT-1.0; IR-1.0) and one-legged stand test (non-dominant leg TRT-0.92; IR-0.97 and dominant leg TRT-0.89; IR-0.92). Excellent to good reliability was shown by 4-step square test (TRT-0.89; IR-0.74), 2-min step in place test (TRT-0.85; IR-0.78), and 30-second chair stand test (TRT-0.86; IR-0.80).

**Conclusion:**

The reliability of individual outcome measures ranged from good to excellent, suggesting that these outcomes have sufficient sensitivity for detecting change with telerehabilitation.

